# Role of the Gut–Brain Axis, Gut Microbial Composition, Diet, and Probiotic Intervention in Parkinson’s Disease

**DOI:** 10.3390/microorganisms10081544

**Published:** 2022-07-29

**Authors:** Subramanian Thangaleela, Bhagavathi Sundaram Sivamaruthi, Periyanaina Kesika, Muruganantham Bharathi, Chaiyavat Chaiyasut

**Affiliations:** 1 Innovation Center for Holistic Health, Nutraceuticals, and Cosmeceuticals, Faculty of Pharmacy, Chiang Mai University, Chiang Mai 50200, Thailand; thangaleela.s@cmu.ac.th (S.T.); kesika.p@cmu.ac.th (P.K.); bharathi.m@cmu.ac.th (M.B.); 2 Office of Research Administration, Chiang Mai University, Chiang Mai 50200, Thailand

**Keywords:** gut microbiota, Parkinson’s disease, gut–brain axis, probiotics, central nervous system, inflammation, neurodegenerative diseases

## Abstract

Parkinson’s disease (PD) is the second-most prevalent neurodegenerative or neuropsychiatric disease, affecting 1% of seniors worldwide. The gut microbiota (GM) is one of the key access controls for most diseases and disorders. Disturbance in the GM creates an imbalance in the function and circulation of metabolites, resulting in unhealthy conditions. Any dysbiosis could affect the function of the gut, consequently disturbing the equilibrium in the intestine, and provoking pro-inflammatory conditions in the gut lumen, which send signals to the central nervous system (CNS) through the vagus enteric nervous system, possibly disturbing the blood–brain barrier. The neuroinflammatory conditions in the brain cause accumulation of α-syn, and progressively develop PD. An important aspect of understanding and treating the disease is access to broad knowledge about the influence of dietary supplements on GM. Probiotics are live microorganisms which, when administered in adequate amounts, confer a health benefit on the host. Probiotic supplementation improves the function of the CNS, and improves the motor and non-motor symptoms of PD. Probiotic supplementation could be an adjuvant therapeutic method to manage PD. This review summarizes the role of GM in health, the GM–brain axis, the pathogenesis of PD, the role of GM and diet in PD, and the influence of probiotic supplementation on PD. The study encourages further detailed clinical trials in PD patients with probiotics, which aids in determining the involvement of GM, intestinal mediators, and neurological mediators in the treatment or management of PD.

## 1. Introduction

Human beings are higher-order organisms living with the microbial world inside and outside of their bodies. Microbes have inhabited the earth for more than a million years before humans [1]. The genome of microorganisms and humans collectively constitutes the human microbiome [2]. The human body is a habitat for various microorganisms such as bacteria, viruses, fungi, and parasites. Almost every place in the human body acts as a distinct microbial niche. The main sites of microbial colonization are the airways, urogenital tract, eyes, skin, oral, pulmonary tract, and gastrointestinal (GI) tract [3,4]. In particular, the human GI tract fosters millions of bacteria, a greater number than that fostered by the eukaryotic cells of the human body [5].

### 1.1. Gut Microbiota

The gut microbiota (GM) has been categorized into autochthonous microbes and allochthonous microbes, with different functions. The former type resides in the epithelial layer of colonic mucosa, whereas the latter one passes through the lumen [6]. The prevalence of GM is not alike everywhere in the GI tract; the distribution varies across the intestinal tract, with preference for three microhabitats: floating cells in the intestinal lumen, adherent cells in the mucosal layer, and intestinal epithelial cells [7]. GM varies from the duodenum to the ileum, with high microbial loads in the colon region [8]. Although there is a great diversity of microbes residing in the human gut, most of the microbiota are from phyla Firmicutes and Bacteroidetes [9]. About 1% of microbes are from other phyla, including Proteobacteria, Actinobacteria, Fusobacteria, Verrucomicrobia [10], Bifidobacteria, Lentisphaerae, and Spirochaetes [11]. The availability of microbes in the GI tract depends on changes in pH, nutrient availability, GI transit, mucin secretion, immune functions, and the host’s age and health [10,12].

The GI tract executes various functions such as digestion of food, absorption of nutrients and water, protection against pathogens, and energy balance, etc. The GM and their metabolites modulate GI functions, such as providing mucosal immune function, intestinal permeability [13], sensitivity [14], enteric nervous system (ENS) activity [15], visceral pain [16]; brain functions and behaviors, including emotions [17], pain [18], and stress responses [19]; and in brain biochemistry [20]. Neural networks control the physiological functions of the GI tract. The autonomic nerves connect the central nervous system (CNS) with the gut [21]. The gut is innervated by the ENS, which is connected to the CNS and allows the exchange of metabolites and information [22].

### 1.2. Gut Microbiota, Neuronal System, and Neurodegenerative Diseases (NDs)

The CNS modulates the GI tract and the ENS through the network of vagus nerves, with sympathetic and parasympathetic nerves of the autonomic nervous system (ANS), hypothalamic–pituitary–adrenal (HPA) axis, gut hormones, and cytokines [23]. The CNS modulates the GM through the enteric environment and signaling molecules [24]. The GM performs various functions such as nutrient metabolism, synthesis of vitamins, breaking down of drugs, promoting intestinal barrier [25] and maintaining the production of short-chain fatty acids (SCFA). During these functions, the GM releases small molecules and metabolites that can stimulate mucus secretion [26]. In addition to these functions, the GM triggers the innate immune system and leads to the development of gut-associated lymphoid tissue (GALT), which results in the development of adaptive local immunity [27].

The function of the nervous system is disturbed if there are any physiological interruptions in the GM, which can be termed gut dysbiosis. Gut dysbiosis can directly modify the function of the immune system and tissue barriers, such as the blood–brain barrier (BBB) [28], which in turn influences the brain functions, and results in stress response, cognitive activities, and changes to behavior and memory [18,20], possibly leading to depression [29] and anxiety [30]. The GI functions depend on the interactions of the gut microbes with the brain, through the creation of a “microbiota–gut–brain axis” network [28]. Studies showed that GM takes part in the physiology of the brain. In a case where there are any interruptions in the microbiota by any harmful nutrients, microbes can elicit different signaling pathways, including oxidative stress, energy metabolism, mitochondrial function, and neuroinflammation, and disturb the epigenetic mechanisms, which eventually influence gene expression [31].

The gut and brain connections are mediated by various microbial metabolites called neuromodulators [28,31]. In addition to maintaining immune and metabolic health [4], the gut–brain axis performs various other functions such as brain development [32] neurogenesis [33], and the CNS–ENS interactions in NDs and neuroinflammation diseases such as Parkinson’s disease (PD), Alzheimer’s disease (AD), multiple sclerosis (MS), autism spectrum disorder (ASD) [34], and major depressive disorders (MDDs) [27].

### 1.3. Parkinson’s Disease (PD)

Among other NDs, patients with PD showed GI disturbance even before developing motor symptoms [35]. About 80% of PD patients are observed with clinical GI symptoms such as nausea, vomiting [36], and constipation [37]. Along with these symptoms, stomach and colon motility disturbances are very common in PD [38]. The GM communicates with the brain and vice versa. Any changes in the gut–brain axis may interfere with the function of each other [39].

The clinical manifestations of PD and the GM–brain axis need to be well characterized, including GI modulations and immunological and neuroendocrine mechanisms. Many extra-neuronal factors, such as nutrition and environmental factors, may cause impacts on metabolism, the immune system, distress to the brain physiology, and neuronal function that leads to neuropathogenesis [31]. Nutritional habits greatly affect GM’s colonization, maturation, and changes throughout human life [40]. The ecology of GM changes according to age, diet, medications, and geographical location [41].

### 1.4. Probiotics

Probiotics are live microorganisms which, when administered in adequate amounts, confer a health benefit on the host [42]. Probiotics could improve the gut microbial composition and host immune system [43]. Gut dysbiosis and dysfunction cause GI diseases, neuropsychiatric disorders, and NDs. Probiotics could improve gut health by improving intestinal integrity, reducing inflammatory responses, and improving the HPA axis and neuroprotective activities [44].

The probiotics maintain the microbes–gut–brain interaction by regulating intestinal neuron excitation, reducing intestinal permeability, microbial translocation, and neuroinflammation [43]. Probiotic intervention could aid in managing the metabolic syndrome, characterized by central obesity, elevated triglycerides, low-density lipoprotein, impaired fasting glucose, and hypertension [45]. The supplementation of probiotics reduced the expression of inflammatory cytokines and TLR-2 expression, and reduced the symptoms of chronic intestinal inflammation, IBD [46,47]. Equally, probiotic supplementation improved the fasting blood glucose level and insulin sensitivity [48,49].

Psychobiotics were reported recently as an effective influencer of the CNS and gut–brain axis [50]. Probiotics are reported to treat and manage neurological conditions such as ASD [51,52]. In conclusion, probiotic supplementation improves gut health and reduces neuroinflammation, improving neurological diseases.

The current review summarizes the role of GM in health, the GM–brain axis, the pathogenesis of PD, the role of GM and diet in PD, and the influence of probiotic supplementation on PD.

## 2. Gut Microbiota of Humans across Ages

Advanced sequencing techniques have revealed that over 1000 species and 7000 bacterial strains were detected in human GM [53]. The human GM, from infancy to childhood, depends on the birth mode, gender, maternal antibiotic usage, breast/formula/mixed feeding, and GI tract symptoms [54]. The age spectrum studies revealed that members of phyla Bacteroidetes, Firmicutes, Proteobacteria, and Actinobacteria were dominant during the first 3 years of life. From birth to 27 months, Bacteroidetes abundance increased and remained stable until 3 years of age. On the contrary, the abundance of Proteobacteria significantly decreased from birth to 2 years of age [55]. Children born through caesarean section showed a predominance of Bacteroidetes members compared to children born by vaginal birth. The studies have revealed that the intestinal microbiota of infants born by vaginal birth resemble their mother’s vaginal microbiota, dominated by *Lactobacillus*, *Prevotella,* and *Sneathia*. In the case of those born by caesarean section, infants’ intestinal microbiota resemble the skin microbiota of mothers, such as *Staphylococcus*, *Corynebacterium*, and *Propionibacterium* [56]. It stated that the delivery mode could influence the occurrence of microbiota and the resulting immunological development in infants [57].

Secondarily, with its rich nutritional, bioactive, and immunological components, breast milk helps the maturation of the newborn intestine and its microbial composition. The presence of lactoferrin, α-lactalbumin, secretory IgA, lysozyme, oligosaccharides, complex lipids, and glycoconjugates in the breast milk renders a protective function in infants. Breastfed infants’ intestines receive protection against the colonization of pathogens by releasing glycomacropeptides. Lactoferrin is an antimicrobial agent in breast milk, protecting against GI and respiratory infections in breastfed infants [58]. The oligosaccharides in the breast milk induce the multiplication of *Bifidobacterium* spp. and *Lactobacillus* spp. The formula milk powder stimulates Enterococci and Enterobacteria [59]. The introduction of solid foods to infants increases and evolves the microbial diversity from infants to an adult-like state. It remains dominated by a few enterotypes such as *Bacteroides*, *Prevotella,* and *Ruminococcus* [60]. The GM of breastfed infants showed a less diverse microbial population; primarily a higher level of *Bifidobacterium* spp. including *B. breve*, *B. bifidum,* and *B. longum* were found, because these organisms survive using human milk oligosaccharides [61,62].

In preterm infants, the gut microbial composition is extremely challenging due to immature organ development, duration of hospital stays, and medications. The GM of preterm infants were reported to contain facultative or opportunistic anaerobes and pathogenic bacteria [4]. The preterm infants showed a high population of the Enterococcaceae family. Initially, the gut of a newborn is a completely aerobic environment. Gradually, it changes into anaerobic [63] and allows the development of anaerobes such as *Bifidobacterium*, *Clostridium,* and *Bacteroides* [64]. The study on the placental microbiome profile revealed that microbial colonization in the human gut starts before birth. Like the human oral microbiome, it was dominated by the members of Firmicutes, Bacteroidetes, Fusobacteria, Proteobacteria, and Tenericutes [65]. The microbiota of infants contains the species from their mothers. There exists clear transmission of the vaginal microbiota of mothers, which can be seen in the meconium of full-term infants. In addition, 30 genera of microbes were found in infants, derived from the amniotic fluid, vagina, and oral cavity [66]. The GM of older children (7–12 years) were rich in *Bifidobacterium* and *Faecalibacterium* spp., whereas adults have *Bacteroides vulgatus* and *Bacteroides xylanisolvens* [67]. Adults were also reported to have genera Bacteroides, Prevotella and Ruminococcus [68]. *Methanobrevibacter smithii* and *Methanosphaera stadmanae* were also higher in children (0–10 years) [69].

The human GM possess extreme inter-individual variations in older people (≥65 years), proved to be functionally relevant, personalized, specific, and found to be stable in healthy adults, but differs from the core microbiota of younger adults [54]. The regression of physiological function due to age development can be called aging. Despite aging progress, the human GM composition affects intestinal transit, eventually altering the gut bacterial ecosystem [70]. In addition to aging, the human GM composition is modulated by other factors such as environment, medications, and diet [40]. Aging indicates a wide range of variation in the microbiota in the human body. Such a decline in microbiota leads to weakening immune function and an increased incidence of infections in the elderly [71]. Bacteroides, Eubacterium, Clostridiaceae, and Enterobacteriaceae were proportionally higher in infants and elders; *Bifidobacterium* was enriched in infants but declined with aging [72,73]. Higher *Enterobacteria* were found in children than adults [74], and Lachnospiraceae were enriched in adults [73]. Organisms from genera *Oxalobacter, Butyrivibrio,* and *Lactobacillus* have been associated with aging, and decreased in extremely elderly subjects. Centenarians are reported to have Prevotellaceae in their gut [75].

**Figure 1:**
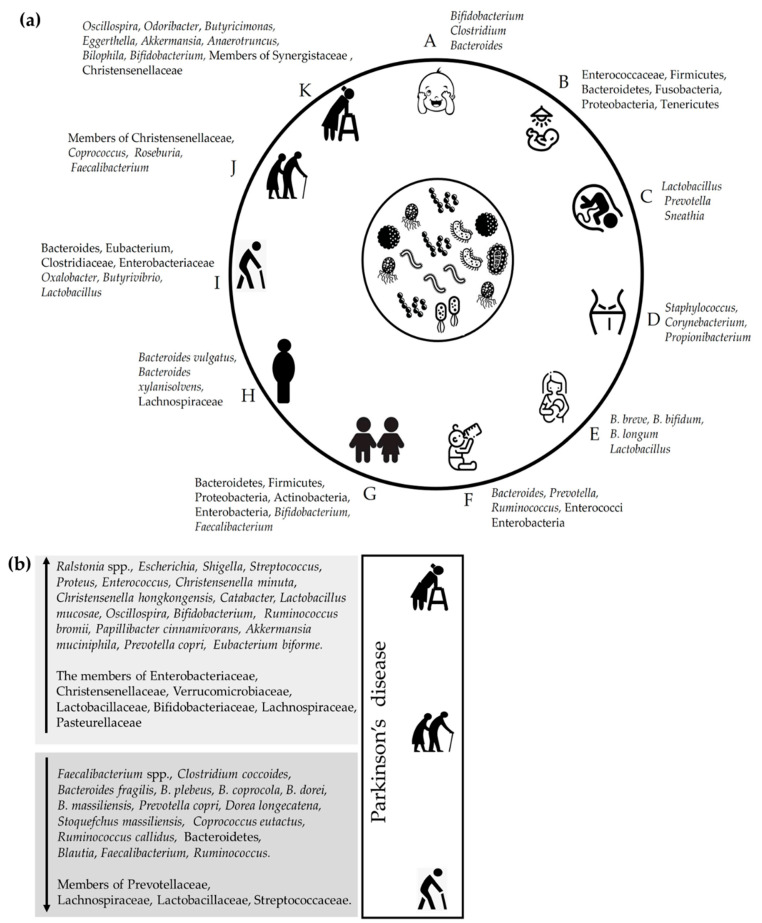
(**a**) Diagrammatic representation of predominant gut microbiome members in humans across ages. A: newborn baby; B: pre-term infants; C: infant from vaginal birth; D: infant from caesarean; E: breastfeeding baby; F: formula-feeding baby; G: children; H: adult; I: Elders; J: centenarians; K: semi-supercentenarians. (**b**) The altered gut microbiome in Parkinson’s disease. ↑ indicates the increase in the abundance of microbes; ↓ indicates the decrease in the abundance of microbes.

A study by Biagi and his colleagues examined the core microbiota among four different age groups, including young (22–48 years), elderly (65–75 years), centenarian (99–104 years), and semi-supercentenarian (105–109 years). The results revealed that the abundance of members of Ruminococcaceae, Lachnospiraceae, and Bacteroidaceae decreases with aging. Extreme longevity showed the invasion of Mogibacteriaceae and Synergistaceae. Members of the family Christensenellaceae, associated with improved renal function, increased in centenarians and semi-centenarians. Other genera, *Akkermansia* and *Bifidobacterium,* are increasingly found in semi-supercentenarians, and found to improve immunomodulation and metabolic homeostasis. The microbial diversity showed the age-related ascending and descending trajectory. Bacteria from genera *Coprococcus*, *Roseburia,* and *Faecalibacterium* were reported in Chinese centenarians, which were negatively associated with aging, and can be a part of aging irrespective of lifestyle and dietary habits. *Oscillospira*, *Odoribacter*, *Butyricimonas*, *Eggerthella*, *Akkermansia*, *Anaerotruncus*, *Bilophila,* and members of families Synergistaceae and Christensenellaceae were positively associated with age in the semi-supercentenarians [76] (Figure 1).

The gut microbes in adults are individual-specific, stable, and strong, because of the absence of external factors such as dietary changes and antibiotic treatment [77]. The gut is relatively dominated by Firmicutes and Bacteroidetes, and less abundantly by Actinobacteria, Proteobacteria, and Verrucomicrobia. Irrespective of gender, nationality, and body mass or age, three genera, *Bacteroides*, *Prevotella,* and *Ruminococcus*, were considered robust and well-defined microbial communities. The intestinal mucin layer of young adults was colonized by *Akkermansia muciniphila* (*A. muciniphila*), which helps in supporting gut barrier integrity, and preventing leakage and inflammations. In the elderly, the gut microbial composition and diversity are reduced because of increased pro-inflammatory commensals and decreased beneficial microbes, such as Verrucomicrobia. This is the reason for gut leakage, inflammation, and aging-related health issues [78]. SCFAs are the vital metabolites of GM, which may reduce inflammation and insulin resistance [79].

The GM composition determines the inflammatory functions in older adults. The metagenomic study with 100 elderly individuals showed that the core microbiota in elders differed from that of young subjects, and the ratio of Bacteroides to Firmicutes varied between individuals. The presence of Proteobacteria, *Bifidobacterium* from Actinobacteria and *Faecalibacterium*, especially anti-inflammatory species *Faecalibacterium prausnitzii* (*F. prausnitzii*), and butyrate-producing *Ruminococcus* sp., were reported in the elderly subjects [80]. In aging, the GM composition changes, and gut dysbiosis increases. The loss of the mucin layer in the gut results in the loss of butyrate-producing commensals such as *Intestinimonas butyriciproducens* (*I. butyriciproducens*), *F. prausnitzii*, *Roseburia faecis* (*R. faecis*), and *Anaerostipes butyraticus* (*A. butyraticus*), which eventually results in gut dysbiosis and gut leakage. Any increase in the release of endotoxin or inflammatory cytokines such as IL-1 and IL-6 (interleukin-1 and 6), interferons (IFNs), and tumor necrosis factor-α (TNF-α) results in gut leakiness, and initiation of inflammatory events and age-associated diseases in the elderly subjects [81]. The bacterial endotoxin is one of the causes of systemic inflammation, and oxidative stress can induce neuroinflammation in the CNS [82].

## 3. Impact of Gut Microbiota on Human Health

The human microbiota is said to be a huge network that plays an indomitable role in the health of humans. With the help of the advancement of recent technologies, the complicated functional role of GM can be excavated gradually [83]. The commensal, symbiotic and pathogenic microbial community residing in the human body can trigger or disturb the host’s energy metabolism and immune functions [84]. The GM act as an essential metabolic organ of humans that tends to digest, absorb, and metabolize the dietary components, which are indigestible for humans, and supports host metabolism [85], resistance to pathogens [86], defense, and detoxification [87], and causes diseases, if any dysbiosis, by influencing the host metabolism [84].

The metagenomic studies showed that GM is associated with various human diseases [88]. Any physiological changes in the GI tract influence the GM and may result in susceptibility to infections [70]. Dietary practices [4] and the phylogeny of the host directly influence the intestinal microbiota in mammals and other species [89]. The high fat, high sugar Western diet changed the microbiota [90]. A human study with high-fat–low-fiber and low-fat–high-fiber diet was also found to cause changes in the GM. Hence, it can be understood that diet can act as an external factor that correlates with the enterotypes of individuals [91]. Enumerating GM is important for studying the characteristics of organisms and the susceptibility of humans to diseases [57].

Any alterations in the GM can lead to immune dysregulation and autoimmune disorders [92]. There exists a dynamic interaction between the GM and the host immune system. GM produces many metabolic products that mediate the signaling between the gut epithelial cells and immune cells. Gut epithelial cells act as a mucosal barrier and separate the GM and the host immune cells, minimizing intestinal permeability. Any impairment in the interaction between GM mucosal immune system may lead to the disruption of the epithelial barrier and increased susceptibility to infections [93].

Gut bacteria such as *F. prausnitzii, R. intestinalis,* and *A. butyraticus* [94] produce SCFA [95]. Members of the *Butyrivibrio* genus were found to produce butyrate and are responsible for the colonic health of humans [96]. *Bifidobacteria* produce acetate that regulates intestinal inflammation [97]. Firmicutes and Bifidobacteriaceae modulate gut homeostasis. Meanwhile, members of facultative anaerobic Enterobacteriaceae are the markers for gut dysbiosis. Propionate and butyrate resist the growth of pathogens in the gut through intracellular cytoplasmic acidification. As a feedback mechanism, to sustain the aversive environment, pathogens such as *Salmonella* and *Shigella* invite neutrophils, reduce the availability of SCFA in the gut epithelium, and sustain their growth in the gut [98,99]. Some commensals inhibit the growth of opportunistic pathogens with the help of SCFA. For instance, *Bifidobacterium* inhibits the colonization of *Escherichia coli* (*E. coli*) [41]. Another restrictive function of commensals Enterobacteriaceae is the utilization of residual oxygen available in the gut, which produces a completely anaerobic environment; these unfavorable anaerobic conditions suppress the virulence of pathogens such as *Shigella flexneri* (*S. flexneri)* [100]. GM is predominantly anaerobes, which prevent the translocation of aerobes and facultative anaerobes in the GI tract. Few organisms, such as *E. coli* and *Bacteroides fragilis* (*B. fragilis*), help synthesize vitamins B1, B2, B5, B6, B12, and K, folic acid, and biotin. *B. fragilis* and *Fusobacterium* spp. degrade xenobiotics and sterols [7].

Dysbiosis is not merely the increase in abundance of potential pathogens, but also includes a lack of commensals in the gut. The gut microbes support the maturation and functioning of the immune system. Dysregulation of the same leads to disease conditions such as immune dysregulation, diabetes, autoimmune diseases, cardiovascular diseases (CVDs), and inflammatory bowel disease (Crohn’s disease) [101], irritable bowel syndrome (IBS) [102], colon cancer [103], diabetes [104], obesity [105], rheumatoid arthritis [106], osteoporosis [107], and gout [108]. The GM of elderly humans differs from that of young and middle-aged adults. The microbial shift during elderly age results in clinical manifestations of age-related pathologies. Gut dysbiosis is the major reason for the premature death of the elderly [78].

## 4. Gut Microbiota and Brain Axis

The gut–brain connection is bidirectional and responsible for connecting the ENS and CNS. The gut–brain axis regulates endocrine, humoral, metabolic, and immune functions. The microorganisms in the GI tract modulate the development and functions of the CNS and ENS [109]. The CNS regulates the GI tract through the ENS via sympathetic and parasympathetic nerves and the HPA axis [110]. The ENS is responsible for initiating and developing intestinal transit and migrating contractions of the intestine. Reduced migrating contractions cause constipation, whereas increased migrating contractions in the gut cause diarrhea-predominant IBS [111]. The migrating gut contractions are associated with the frequency of food intake, sleep, stress, and GM composition. The ANS controls the mucus secretion, size, and quality of the mucus layer in the gut and the gut epithelial cells [112].

The intestinal epithelial cell regulates the gut immune cells, and induces an immune response in the intestinal mucosa [112]. The secretion of neuroendocrine molecules, such as catecholamines, serotonin, dynorphin, γ-aminobutyric acid (GABA), and cytokines released into the gut lumen by the neurons and enterochromaffin cells, are modulated by the CNS [113]. Thus, synthesized catecholamines such as norepinephrine enhance the pathogenic activities of *Campylobacter jejuni* (*C. jejuni*) [114], as well as the proliferation of other enteric strains [115]. The two-way microbe-to-host signaling is carried out through the ENS and the brain. The receptors of the host cells receive information via signaling molecules such as SCFA, GABA, tryptophan precursors, serotonin, cytokines, and catecholamines [111]. The connections stay connected in neurocrine and endocrine pathways with the help of vagal afferents and receptors in the brain [116].

The association between GM and brain pathology necessitates diagnosing, managing, and treating neurological disorders. Still, the link between GM alterations and brain disorders poses many unanswered questions [110]. Any changes in the GM–brain axis result in altered gut microbiota and brain functions. GM is subjected to changes during stress and results in the release of cytokines and stress-induced immunoenhancement. Bailey et al. analyzed microbiota stability and bacterial translocation in a mice model. The results showed that the abundance of *Bacteroides* was reduced, and the abundance of *Clostridium* was increased. Other bacterial genera such as *Coprococcus*, *Pseudobutyrivibrio,* and *Dorea* were also significantly changed due to stress-induced release of IL-6 and monocyte chemoattractant protein-1 (MCP-1) [117].

## 5. How Does the Gut–Brain Bidirectional Pathway Work?

Various pathways link the gut and brain together. The bond exists between the ENS, vagus nerve, immune system, and gut microbes [5]. The nerves innervate the GI tract from the CNS, ENS, and ANS, and the gut system is regulated by four levels of nervous regulation [118]. The first level of regulation is the ENS, composed of myenteric and submucosal nerve plexuses, and the motor and sensory neurons, to carry out different impulses and information. The second level of regulation depends on the prevertebral ganglia, which transmits signals from the ENS and CNS nerves. The third level of regulation is the CNS, which gathers signals from various brain centers and the spinal cord, and transmits that information collected from internal and external sources to the ENS, ANS, and neuroendocrine system, which results in the regulation of smooth muscles, glands, and blood vessels. The fourth level of regulation involves the highly advanced brain regions such as cortical, subcortical, brain stem and basal ganglia. The information from the cortical regions reaches the brain stem nuclei from basal ganglia, and hence forms the neuroendocrine network, which tends to connect the GI tract and CNS at various levels, and comprises the gut–brain axis. Hence, in any way, a small disorder in the line of gut–brain axis connections leads to dysfunction of the gut and brain [119]. One of the intriguing phenomena of GM is the regulation of adult hippocampal neurogenesis. The adult hippocampus is important for learning and memory; once the hippocampal neurogenesis becomes affected due to gut dysbiosis, it may lead to neuropathogenesis and cognitive declines [120].

**Figure 2:**
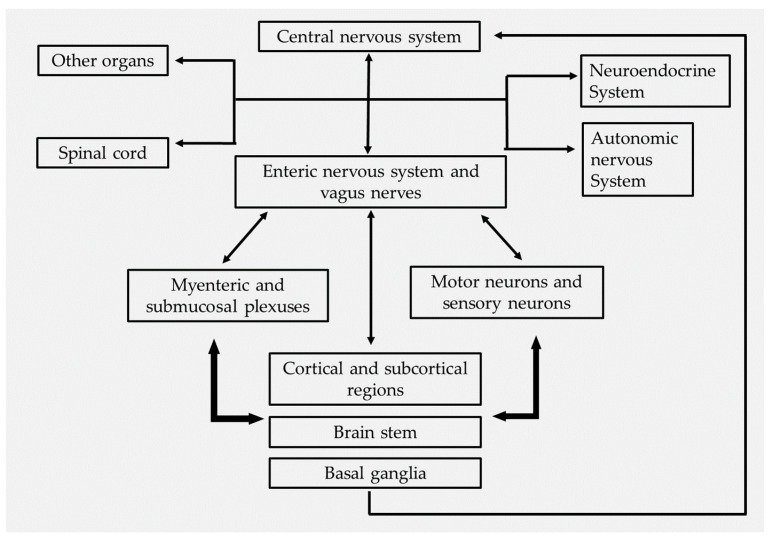
Bidirectional communication and the mediators of the gut–brain axis.

The vagus nerve helps transmit peripheral immune signals to the CNS and controls the heart and gut motility. A mouse vagotomy study revealed that the gut microbes directly regulate the brain through vagus nerves, and no behavior change was observed in the mice after vagotomy [121]. The GM can modulate the metabolites such as SCFA, circulating tryptophan, serotonin, BBB permeability, and immune cells of the gut and brain; thus, it involves neuropsychiatric disorders. SCFAs were involved in NDs, neurodevelopment, and cognitive function [20]. Specific neurochemical changes were observed in autism-related characteristics in rats injected with propionic acid [122]. The biochemical signaling carried over by the gut and brain becomes affected during the dysbiosis, resulting in the activation of the immune response, leading to the secretion of inflammatory substances and triggering a wide range of psychiatric diseases [123] (Figure 2).

## 6. Pathophysiology of Parkinson’s Disease (PD)

The etiology of PD is multifaceted, including genetics, aging, gut dysfunction, and environment [124]. Familial PD can occur because of the point mutations in the alpha-synuclein (α-syn) gene, and locus duplication, triplication and sporadic PD may be due to genetic and environmental factors. Pesticides such as rotenone, paraquat, and 1-methyl-4-fenyl 1,2,3,6-tetrahydropyridine are one of the causative agents of PD [124].

James Parkinson first described PD in his “An Essay on the Shaking Palsy” in 1817. He originally described the muscular weakness and non-tremulous form of PD [125]. Many decades later, after Charlot’s study in 1957, the cause of PD was recognized as a loss of neuronal cells in substantia nigra [126]. In 1960, the neurotransmitter dopamine was found to be diminished in the striatum of PD patients [127]. The other contributing factors for the fall of dopaminergic and non-dopaminergic neurons in the brains of PD patients include misfolded proteins, ubiquitin-proteosome, and autophagy lysosomal system errors, increased oxidative stress, mitochondrial dysfunction, and inflammation [128,129].

PD affects several brain regions, including pigmented nuclei in the midbrain, brainstem, olfactory tubercle, cerebral cortex, and some of the peripheral nervous system [130]. The degeneration of dopaminergic neurons of the substantia nigra compacta (SNc) region and their projections to the striatum developed during the disease progression; due to this degeneration of neurons of limbic portions of the striatum, the motor signs of PD are visible before the non-motor signs [131]. In addition to the loss of dopaminergic neurons, serotonergic cells in the median raphe, noradrenergic cells in the locus coeruleus, and cholinergic cells of the nucleus basalis are also involved to a lesser extent.

Most PD is idiopathic, dominantly inherited genetic variants associated with intraneuronal Lewy bodies inclusions. Some other genetic causes for PD, such as autosomal-dominant and recessive susceptibility genes, have now been identified. Mutations in the α-syn gene were the first identified genetic cause of PD. Other most common mutations are in the gene glucocerebrosidase (GBA) and the lysine-rich repeat kinase 2 (LRRK-2) gene [132]. α-syn is involved in the mitochondrial function and synaptic plasticity, highly concentrated in the nerve terminals and abnormally aggregated as Lewy bodies, which are the prominent component of PD [132]. Impaired proteasomal degradation of ubiquitin-C-terminal hydrolase-L1 and β-glucocerebrosidase [133,134] results in intraneuronal accumulation, misfolding, and phosphorylation at serine-129 of α-syn [135]. The toxic aggregation of α-syn affects the nigrostriatal dopaminergic neurons and induces dopamine autotoxicity [136].

The studies showed that the α-syn aggregation could be initiated in the gut and olfactory bulb [137]; within the GI tract, the deposited α-syn showed a rostro-caudal gradient and was highly concentrated in the submandibular gland and lower in the esophagus [138]. Later, the vagus nerve spreads the α-syn to the brain stem and toward the cortex [137]. Thus, α-synucleinopathy causes neurodegeneration, impaired axonal transport, and degradation of synaptic terminals [139]. A recent study showed the involvement of microRNAs (miRNAs) in PD pathogenesis [140]. Parkin, DJ-1, PINK1, ATP13A2, DNAJC6, PLA2G6, SYNJ1, FBOX7, SNCA, LRRK2, and VPS35 are considered risk factors for PD [141,142,143,144,145,146,147]. Endogenous toxins such as misfolded or aggregated proteins, synuclein and *tau* [148,149], and pro-inflammatory cytokines secreted by T lymphocytes and glial cells, are also associated with PD pathogenesis [150,151].

The four important clinical features of parkinsonism are TRAP: tremor at rest, rigidity, akinesia (bradykinesia), and postural instability [152]. Bradykinesia is an easily recognizable symptom of PD, observed with decreased neuronal density in the substantia nigra, which results in disturbed motor activities [153,154]. Tremor is one of the most common PD symptoms and involves involuntary movement of hands, lips, chin, jaw, and legs [155]. Clinical-pathological studies revealed that the neurons in the subgroup of the midbrain degenerated in PD patients with tremors [156]. The next cardinal symptom is rigidity, characterized by resistance and pain [157,158]. PD patients may also show postural deformities with flexed neck, trunk, elbows, and knees [159,160]. Some skeletal deformities such as neck flexion, truncal flexion, scoliosis [161,162], and extreme flexion of the thoracolumbar spine (camptocormia) are also common in PD [162]. The late stage of PD is characterized by postural instability, which may cause hip fractures [163]. Freezing, which is akinesia, means the complete loss of movement [164], affects the legs, arms, and eyelids [165], and is reported more frequently in men than in women [166]. In addition, some of the non-motor symptoms such as autonomic dysfunction, cognitive disorders, and sleep abnormalities are common features of PD [156].

The bidirectional communication between gut and brain in the case of PD is represented by an integrative organization of both intrinsic and extrinsic nervous systems [167]. PD shows GI dysfunction and cardiovascular, urogenital, thermoregulatory, sleep, and respiratory abnormalities [168]. The PD lesions initiated in the ENS later involve the CNS or vice versa. α-syn lesions are transmitted to the midbrain via the nasal olfactory lobe, and to the temporal lobe and GI system via the ENS [169].

**Figure 3:**
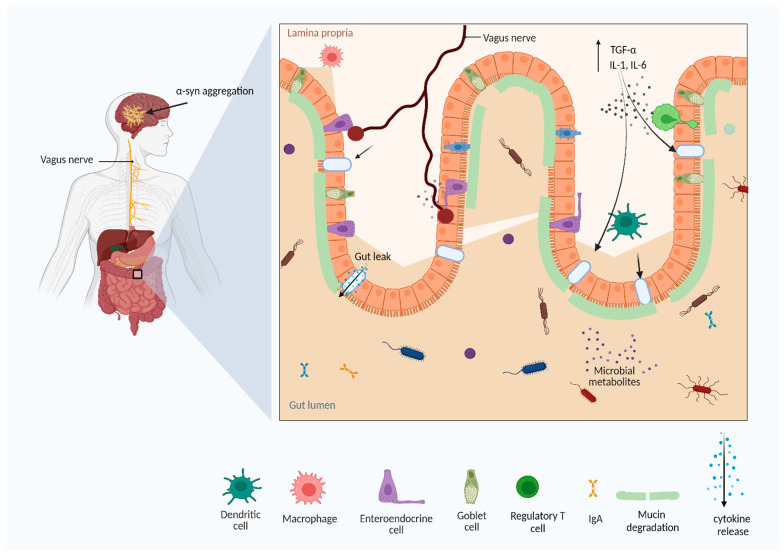
The gut dysbiosis and Parkinson’s disease. Gut dysbiosis and defects in intestinal barrier function facilitate the release of material metabolites, endotoxins, and other antigens into the gastrointestinal system, which further activates the immune system and the release of pro-inflammatory cytokines. Chronic immune activation may cause neuroinflammation and neurodegenerative diseases (Figure created using BioRender.com).

According to Braak’s hypothesis, pathogens may enter through the oral, nasal, or digestive tract, and reach the gut, initiating Lewy bodies in case of sporadic PD [124]. Then, Lewy bodies occur in the dorsal motor nucleus (DMN) of the vagus in the medulla oblongata, vagus nerves, and anterior olfactory nucleus. The Lewy bodies spread within the CNS, substantia nigra, locus coeruleus, neocortex, mesocortex, and prefrontal cortex [170] (Figure 3).

### 6.1. Toll-Like Receptors Expression in PD

Toll-like receptors (TLRs) belong to the pattern recognition receptors family [171]. TLRs are associated with activating the immune cells and initiating the immune response. Different types of T and B lymphocytes were found to express a variety of TLRs [172]. TLRs recognize antigens and cause immune responses through antigen-presenting cells. TLRs were also found to regulate the functions of CD4+ and CD25+ Treg cells (regulatory T cells) and modulate the immune response [173]. In the case of gut dysbiosis, GM trigger inflammatory effects against Lewy bodies and its derived antigens via CD4^+^ T-cell response, causing early gut inflammation, which progresses to PD [174]. In PD, the hyperphosphorylated α-syn aggregates in the brain [175] stimulate innate immune responses in the microglial cells [176] by increasing the TLRs expression in the glial cells [177], especially in the substantia nigra region [178]. TLRs can act as lipopolysaccharides (LPS) receptors, a cell-wall component of gram-negative bacteria. It binds the LPS and activates the pro-inflammatory and anti-microbial cytokines [179].

**Figure 4:**
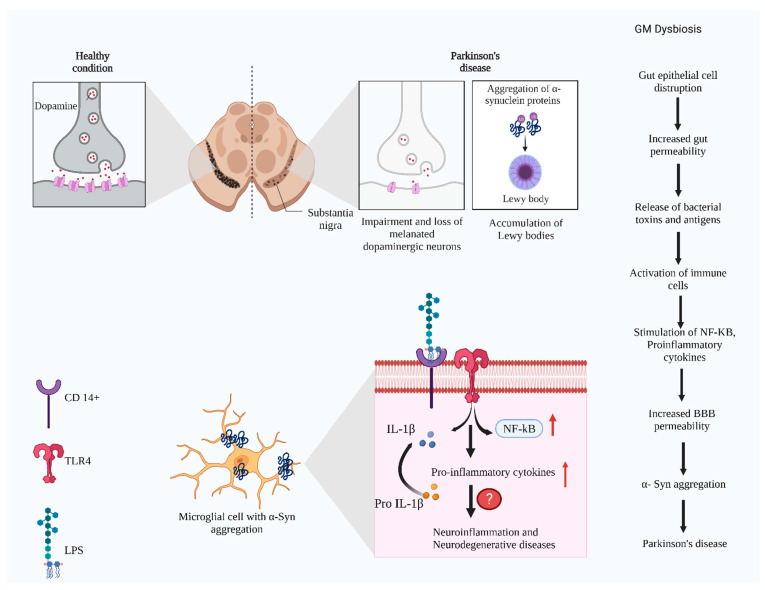
The representation of molecular signaling and progression of Parkinson’s disease in substantia nigra. Gut dysbiosis activates the immune system. The released cytokines may disturb the blood–brain barrier, facilitating the entry of bacterial metabolites and other antigens to the central nervous system. It causes α-syn aggregation in the substantia nigra of the brain. Hyperphosphorylated α-syn recruits the TLR4 and CD14+ in the microglial cells, promoting the neuronal immune responses by activating the NF-κb, pro-IL β pro-inflammatory cytokines. The exact mechanism and the players are not elucidated completely (Figure created using BioRender.com).

Higher TLRs expression was found in the brain of α-synucleinopathies such as PD, dementia with Lewy bodies, and multiple system atrophy, and is involved in the innate immunity, which becomes a therapeutic target for these disorders [180]. TLRs’ activation causes neuroinflammation, stimulates NF-κB and pro-IL-1β, and triggers NDs [181]. TLR2 and TLR4 are the target for PD, as both these receptors are involved in PD development [182]. Aggregated α-syn can bind to the TLR2 and TLR4 and initiate the immune responses in PD. TLR2 can bind with the oligomeric α-syn, resulting in pro-inflammatory signals and neurodegeneration [183]. TLR4 binds with any monomeric or oligomeric α-syn and helps in clearing the α-syn, thereby exerting an alternative role of neuroprotection [184]. α-synucleinopathy studies in rat models evaluated that blocking TLR2 and activating TLR4 provide protective functions [185,186] (Figure 4).

## 7. Signaling Pathways Associated with Gut Microbial Changes and PD

**Figure 5:**
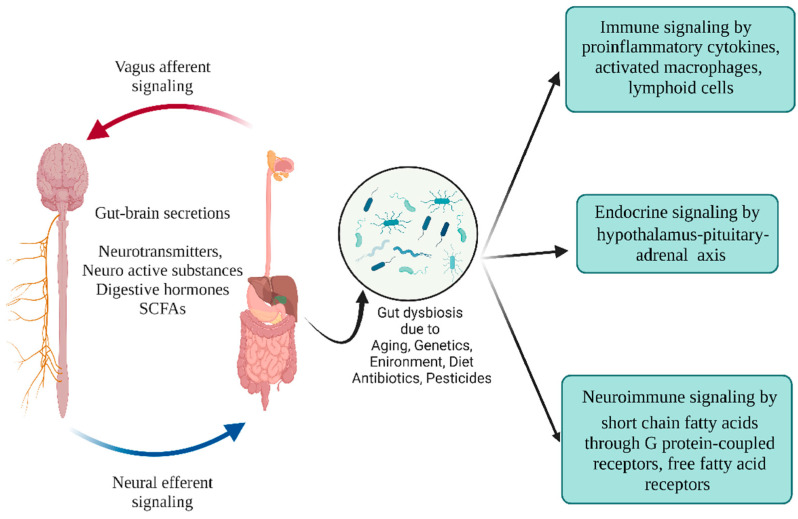
Schematic illustration of endocrine, immune, and neuroimmune signaling pathways. Gut microbes help maintain intestinal integrity by balancing the microbial products, neurotransmitters, and SCFAs across the enteric and immune systems. Microbial dysbiosis triggers activated immune cells, macrophages, dendritic cells, and pro-inflammatory cytokines. (Figure created using BioRender.com).

The GM and the CNS are linked through neural, endocrine, and immune signaling. For example, the microbiota in the gut can induce the cells to synthesize neurotransmitters and digestive hormones, which could alter the brain and behavior [187]. In turn, the CNS can control the GM through adrenergic nerve signaling by regulating the neurotransmitters on immune mediators that shape the GM [187]. Microbiota could affect the hypothalamus-pituitary-adrenal (HPA) axis [188]. Another important metabolic mediator of neuroimmune function are the SCFAs. SCFAs regulate the CNS by modulating microglia [189]. SCFAs signal through the G protein-coupled receptors (GPR), free fatty acid receptors (FFAR), and GPR109. The GPR109 receptor binds to butyrate and induces the production of IL-10-secreting Treg cells [190]. PD patients reported a low abundance of *Prevotella* and SCFA-producing *F. prausnitzii* and *Clostridium* IV, and lowered SCFA metabolites in their gut [191,192,193,194]. A study in α-syn-overexpressing mice demonstrated that the microbial products could be involved in the pathogenesis of PD, by regulating the immune cells in the brain [195]. GM play a prominent role in amino acid metabolism, influencing neuroinflammatory diseases [196]. The intestinal microbiota mediate the endocrine, immune, and neural pathways. Accordingly, gut dysbiosis can alter behavior, mood, and neuroinflammatory responses through the HPA axis, lipopolysaccharide, neurotransmitter, and SCFAs [196] (Figure 5).

## 8. Gut Microbiota (GM) and Parkinson’s Disease (PD)

GI dysfunction has been recognized as associated with PD pathogenesis [197]. GM and its metabolites interfere with the host’s behavior, immunity, cognition, and metabolism [198,199,200,201]. The changes in the GM composition and its metabolites have been identified as a vital reason for the induction and progression of PD [202]. The bidirectional communication between the gut–brain axis is possible through GM [203]. The afferent fibers from the gut are connected to the anterior/posterior, cingulate, cerebral, amygdalar, and insular cortices, and the efferent fibers project to the gut’s smooth muscles [203]. The studies showed that the GM of PD patients were rich in Enterobacteriaceae, which was related to the severity of postural instability and gait difficulties (PIGD) in PD patients, which indicates a positive correlation with PIGD [191].

In the meantime, the abundance of Lactobacillaceae and the decreased abundance of Prevotellaceae in the gut are related to reducing the intestinal hormone ghrelin, a regulating component of nigrostriatal dopamine (DA) [204]. GM regulated the synthesis of DA by controlling the DA-producing enzymes [205]. The majority of DA was produced by the GM, for example, *Bacillus* spp. [18].

Members of Prevotellaceae are reduced in PD patients, which might reduce the mucin synthesis in the gut mucosal layer. The reduction of mucin leads to increased gut permeability, simplifying the entry of bacterial toxins and antigens, thus favoring the aggregation of α-syn in the colon and brain [169,206]. Another possibility of α-syn accumulation is decreased butyrate synthesizing bacteria and increased pro-inflammatory Proteobacteria, which trigger inflammation-induced α-syn misfolding [192]. *Clostridium IV*, *Clostridium XVIII*, *Holdemania*, *Aquabacterium*, *Sphingomonas*, *Butyricicoccus*, and *Anerotruncus* were found in the feces of PD patients and are negatively associated with the disease duration [207]. A reduction in the abundance of Prevotellaceae, Lachnospiraceae, Lactobacillaceae, and Streptococcaceae was reported in PD patients [208].

PD patients are reported to have enteric problems such as bacterial growth in the small intestine, especially *Helicobacter pylori* infection, and constipation. *H. pylori* infection increases the risk of PD and worsens motor symptoms [209]. *Faecalibacterium* spp. level was reduced, *Ralstonia* spp. were significantly increased, and there was no change in the *Bifidobacterium* in the mucus of PD patients [210].

The abundance of Enterobacteriaceae, *Lactobacillus*, *Escherichia*, *Shigella*, *Streptococcus*, *Proteus*, and *Enterococcus* were increased, while *Clostridium coccoides*, *Bacteroides fragilis*, Bacteroidetes, and Prevotellaceae were decreased in Chinese PD patients. In addition, the cellulose degraders *Blautia*, *Faecalibacterium,* and *Ruminococcus* were significantly decreased in PD patients compared to healthy controls [193,194,211]. GM dysbiosis reduces mucin production and increases intestinal permeability, related to PD progression and development [151].

Another study in a mouse model of PD revealed that the hydrogen sulfide produced by *Prevotella* protects the dopaminergic neurons [212]. Dysbiosis of gut microbes reduces the SCFAs, which possibly induces changes in the GI motility and the ENS [193], and increases the neurotoxin and endotoxin production, which can eventually lead to PD development [211]. GM metabolism is responsible for the changes in β-glucuronate and tryptophan degradation pathways in PD [213].

A high abundance of Akkermansia, Bifidobacterium, Ruminococcaceae, and Lactobacillus were found in the fecal microbiome of PD patients, which increases the xenobiotic degrading pathways in PD [214]. Cellulose-degrading bacterial genera such as *Blautia*, *Ruminococcus,* and *Faecalibacterium,* and pathogens *Streptococcus*, *Escherichia*, *Shigella*, *Enterococcus,* and *Proteus* were increased in PD subjects compared to normal subjects [211]. In contrast, Qian et al. reported that *Bacteroides plebeus* (*B. plebeus*), *B. coprocola*, *B. dorei*, *B. massiliensis*, *P. copri*, *Dorea longecatena*, *Faecalibacterium*, *Stoquefchus massiliensis*, *Coprococcus eutactus,* and *Ruminococcus callidus* abundances were decreased. Members such as *Christensenella minuta*, *Christensenella hongkongensis*, *Catabacter*, *Lactobacillus mucosae*, *Oscillospira*, *Bifidobacterium*, *Ruminococcus bromii*, and *Papillibacter cinnamivorans* richness were increased in Chinese PD patients [215]. The members of Christensenellaceae, Verrucomicrobiaceae, Lactobacillaceae, Bifidobacteriaceae, Lachnospiraceae, and Pasteurellaceae were abundantly found in Russian PD patients [216]. The increase in abundances of *A. muciniphila*, *P. copri,* and *Eubacterium biforme* was observed in American PD patients [217]. The bacterial genera responsible for anti-inflammatory and neuroprotective effects (*Butyrivibrio, Pseudobutyvibrio*, *Coprococcus,* and *Blautia*) were reduced in Italian PD patients [215]. Relative abundances of anti-inflammatory butyrate-producing bacteria (*Blautia*, *Coprococcus*, *Roseburia,* and *Faecalibacterium*) were decreased, and the pro-inflammatory bacteria (*Ralstonia*) were found to increase in PD patients [192]. GI complications, dysbiosis, immune dysregulation, and inflammation were found in PD and inflammatory bowel diseases [218,219]. PD patients are often diagnosed with enteric problems such as constipation, and are reported to have less abundance of *Bifidobacterium*, *Prevotella*, and *Lactobacillus*, and are rich in Firmicutes [220].

Aging is a physiological function [221] that induces an imbalance in the pro-inflammatory and anti-inflammatory changes and has been a major factor in various human diseases [222]. Aging reduces the abundance of *Bifidobacteria*, *Lactobacilli,* and SCFA-producing *Faecalibacterium prausnitzii*, *Eubacterium* spp., *Roseburia* spp., and *Ruminococcus* spp. [223]. Aging-induced microbiota changes before the gut dysfunction could disturb various signaling pathways through the microbial metabolites and can negatively affect neurodegeneration [224]. The accumulation of aging-related somatic damages and impaired cellular repair mechanisms can develop PD. The compensatory cellular repair mechanisms, such as mitochondrial oxygenation, ubiquitination, proteolysis, and autophagy processes were reduced during aging, resulting in increased radical production, oxidative stress, genomic instability, and DNA mutations [225], leading to abnormal deposition of brain proteins [226]. Aging can cause axial impairment of gait and postural control in PD [227].

## 9. Role of Diet in Parkinson’s Disease (PD) Incidence and Progression

Studies proved that diet plays a main role in developing neuropsychiatric diseases. Western diet (WD) is a high energy-dense diet with high calorie, high saturated omega-6 fatty acids and fiber, and it is one of the risk factors for PD [228,229]. In another way, the Mediterranean diet (MD) is mainly composed of fresh vegetables, fresh fruits, nuts, seeds, non-dried fish, olive oil, wine, coconut, herbs, and spices, which are associated with a lower risk of developing PD [230]. Diet impacts GM through different perspectives, including food components such as vitamins and fat, which modulate PD’s progression [231].

The GM indirectly controls the development of PD with the help of dietary components. The coffee and caffeine content in the diet is also linked with a decreased risk of PD [232]. Studies found that the consumption of caffeine has protective effects against PD. Chlorogenic acid in coffee inhibits inflammasome, and their polyphenols possess neuroprotective effects and support healthy metabolism [233]. The high consumption of milk and milk products may pose a risk for PD [234]. However, the overall studies did not warrant the avoidance of dairy products. Pesticides, rarely found in milk, are also in the queue to develop PD, but there is no data to support this cause. Exposure to neurotoxins from diet and environment promotes NDs [235]. Moderate and heavy alcohol consumption showed no effects on PD incidence [236]. On the other hand, alcohol could cause intestinal leakiness and disturb the GM, leading to inflammatory responses, and later increasing the risk for PD [237]. Diet could rapidly affect the composition of GM, and modulate the host immune functions, which can trigger intestinal barrier dysfunction, leading to systemic and neuroinflammation, eventually causing NDs.

## 10. Probiotic Supplementation and Parkinson’s Disease (PD)

GM plays a major role in host health and diseases. Gut dysbiosis is the major pathological reason for several illnesses. The GM can be improved through probiotic interventions. The reports on the possible mechanism of probiotics-based adjuvant therapy for PD are limited. Several studies explain the potential role of probiotics or probiotics-containing foods, especially fermented foods, in managing PD. Probiotic *B. longum* subsp. *Infantis* decrease the LPS in the human colonic microbiota in vitro model. Therefore, probiotic supplementation may reduce inflammation [238]. Similarly, fermented milk containing lactic acid bacteria reduced LPS-dependent neuroinflammation and reversed the memory deficits in the mice model [239]. Probiotics Lactobacilli and Bifidobacteria produce antioxidants and vitamins that reduce oxidative stress [240]. Thus, the supplementation of probiotics could improve the antioxidant system and regulate the neuroinflammation of the host.

Enteric infections by *H. pylori* have acted as triggering events for PD and worsened motor symptoms. *H. pylori* infection increases neuropathological conditions, possibly due to the reduced absorption of L-DOPA [241]. The probiotic supplementation of *B. bifidum* CECT 7366 inhibits *H. pylori* infection in vitro and in vivo [242]. Synucleinopathy studies, using *Caenorhabditis elegans*, suggested that the probiotic intervention inhibits α-syn accumulation [243]. Similarly, the probiotic strain *Bacillus subtilis* PXN21 also inhibits α-syn aggregation in *C. elegans* [244].

Probiotics supplementation improves gut health and regulates the host immune system. Probiotics positively modulate brain functions, including anxiety, depression-like behavior, and the gut–brain axis [245]. Probiotics strains such as *Lactobacillus* spp., *Saccharomyces* spp., and *Bifidobacterium* spp. could enhance the epithelial integrity of the intestine, inhibit bacterial growth, and regulate the mucosal immune system; thereby, probiotics aid in managing inflammatory and neuropsychiatric diseases, CVDs, and multiple GI disorders [246,247].

Probiotic supplements may improve PD conditions by altering the PD-associated microbiota, and enhance gut function by reducing intestinal leakage and neuroinflammation in ENS. The supplementation of fermented milk containing *Lactobacillus casei* improves GI discomfort in PD patients. In detail, consuming milk containing *L. casei* for 5 weeks improved stool consistency and reduced bloating and abdominal pain [248]. Similarly, the 3-month supplementation of probiotic tablets containing *Lactobacillus acidophilus* and *Bifidobacterium infantis* reduced abdominal pain and bloating in PD patients [249].

The supplementation of probiotic mix (8 × 10^9^ CFU per day; *Lactobacillus acidophilus*, *Bifidobacterium bifidum*, *Lactobacillus reuteri*, and *Lactobacillus fermentum*) for 12 weeks improved the metabolic profiles and Movement Disorders Society-Unified Parkinson’s Disease Rating Scale (MDS-UPDRS). The probiotic supplementation reduced the high-sensitivity C-reactive protein and malondialdehyde level and increased the blood glutathione compared to placebo. The probiotic-supplemented group recorded the reduction in insulin level, homeostatic model assessment for insulin resistance (HOMA-IR), and quantitative insulin sensitivity check index (QUICKI) scores. In addition, the probiotic supplementation reduced the triglycerides and very low-density lipoprotein-cholesterol levels in PD patients. The results showed that probiotic supplementation could improve the anti-inflammatory and antioxidant systems of PD patients [250]. The supplementation of probiotics *L. salivarius* LS01 and *L. acidophilus* reduced the pro-inflammatory and anti-inflammatory cytokines in PD patients [251].

Ibrahim et al. studied the effectiveness of Hexbio^®^ (A mixture of *L. acidophilus* BCMC^®^ 12130, *L. casei* BCMC^®^ 12313, *L. lactis* BCMC^®^ 12451, BCMC^®^ 02290, *B. infantis* BCMC^®^ 02129, and *B. longum* BCMC^®^ 02120) in PD patients with constipation. In detail, a total of 30 × 10^9^ CFU per day probiotic cells were supplemented in the patients along with 2% fructooligosaccharides (FOS) for 8 weeks. The bowel opening frequency (BOF) was increased in the probiotic supplemented group, and the higher BOF was in the patients in the probiotic group compared to placebo. At the same time, gut transit time was significantly reduced. No significant changes were observed on the Non-Motor Symptoms Scale, Parkinson’s disease Questionnaire 39 summary indices for quality of life assessment, or MDS-UPDRS scores between treatment and placebo groups [252]. Similarly, the supplementation of the probiotic mixture (*L. rhamnosus GG*, *L. acidophilus*, *L. plantarum*, *L. paracasei*, *L. delbrueckii* subsp. *bulgaricus*, and *Bifidobacterium;* 250 × 10^9^ CFU per day) and prebiotic fiber (125 g) for 4 weeks improved the complete bowel movements and bowel frequency in PD patients with Rome III–confirmed constipation [253].

A randomized, placebo-controlled, double-blind trial was carried out on PD patients. A probiotic capsule containing *B. bifidum*, *L. acidophilus*, *L. reuteri*, and *L. fermentum* (8 × 10^9^ CFU per day) was supplemented for 12 weeks. The probiotic supplemented group showed a reduction in the expression of IL-1, IL-8, TNF-α, and an increase in TGF-β, peroxisome proliferator-activated receptor gamma (PPAR-γ), compared to the placebo group. There was no change in the expression of low-density lipoprotein receptors and vascular endothelial growth factors [254] (Table 1).

Probiotic-based treatments could help to treat GI discomforts and PD symptoms. Thus, probiotic-based food supplements can be used to manage PD [259]. The studies on the influences of probiotics on the health status of PD patients are very limited. Further detailed studies are required to determine the beneficial effects of probiotics in PD management.

## 11. Conclusions

It is well known that the gut–brain axis is involved in the incidence and development of various NDs. The evidence revealed that microbial dysbiosis is greatly associated with the incidence of PD. The challenges in understanding the functional correlation between GM and PD must be elucidated by determining microbial roles in initiating the disease and progression. The literature review suggested that an increase and decrease in the relative abundance of certain microbes in the GI tract tremendously changes gut functions. Specifically, the members of Prevotellaceae, Lachnospiraceae, Lactobacillaceae and Streptococcaceae families were less abundant, while the members of the Enterobacteriaceae family were rich in PD patients. PD accompanied by gut dysbiosis can be managed using diet improvements, especially by probiotic supplementations. Understanding the biomarkers of gut dysfunction, its association to PD, and their background cellular mechanisms could be early detection and treatment strategies. Comprehensive studies on personalized PD risk management and its relation to diet, and the development of neuroprotective probiotics (psychobiotics) may support PD management. Extensive clinical trials to validate the various interventions and treatment procedures are needed to explore the direct evidence of the role of GM and PD, which could help to develop therapeutic procedures to treat and manage PD conditions.

## Figures and Tables

**Table 1:** The impact of probiotic supplementation on PD patients and model system.

Study Subjects/Model	Age	No. of Subjects	Probiotic Strain	Dose and Duration	Results	Ref.
*Caenorhabditis elegans* (synucleinopathy model)	-	-	*Bacillus subtilis* PXN21	*C. elegans* fed on *B. subtilis* PXN21	The α-syn aggregation was suppressed. Genes involved in sphingolipid metabolism show altered expression (*lagr-1* and *asm-3* were up-regulated; *sptl-3* was down-regulated), which prevents the α-syn aggregation.	[244]
PD patients with chronic constipation	-	*n* = 40	*Lactobacillus casei Shirota*	65 mL of fermented milk per day (6.5 × 10^9^ CFU) for 6 weeks.	The stool consistency and bowel habits were improved	[248]
PD patients with GI-NMS	76.05 ± 2.09 years	*n* = 40 (*n* = 20 received probiotics; *n* = 20 received trimebutine)	*Lactobacillus acidophilus* and *Bifidobacterium* *infantis*	60 mg per tablet; One tablet twice per day for 3 months.	The abdominal pain and bloating were improved	[249]
PD patients with movement disorders	50–90 years	*n* = 60 (*n* = 30 received probiotics; *n* = 30 placebo)	*L. acidophilus*, *B. bifidum*, *L. reuteri*, *L. fermentum*	(2 × 10^9^ CFU for each strain) 8 × 10^9^ CFU per capsule; One capsule per day for 12 weeks.	MDS-UPDRS was decreased. hsCRP, MDA, and GSH levels were reduced. Insulin level and insulin resistance were reduced. Insulin sensitivity was increased.	[250]
PBMCs were isolated from PD patients.	70 ± 8 years	*n* = 40	*L. salivarius* LS01, *L. plantarum* LP01, *L. acidophilus* LA02, *L. rhamnosus* LR06, *B. breve* BR03, *B. animalis* subsp. *lactis* BS01	1 × 10^6^ cells/plate of PBMCs treated with probiotic strains in 1:1 ratio for 24h	The pro-inflammatory cytokines (TNF-α, IL-17A, and IL-6), oxidative stress levels, and growth of pathogens (*Escherichia coli*, *Klebsiella pneumoniae*) were decreased.	[251]
PD patients with constipation (ROME III criteria)	50–80 years	*n* = 48 (*n* = 22 received probiotics and FOS; *n* = 26 placebo)	Hexbio^®^ (*L. acidophilus*, *L. casei*, *L. lactis*, *B. infantis*, *B. longum*)	107 mg of each strain (30 × 10^9^ CFU) and 2% FOS and lactose; twice daily for 8 weeks	The bowel opening frequency and whole gut transit time were improved.	[252]
PD patients with constipation (ROME III criteria)	Experimental group: 71.8 ± 7.7 years; placebo group: 69.5 ± 10.3 years	*n* = 120 (*n* = 80 received fermented milk; *n* = 40 placebo)	*L. rhamnosus GG*, *L. acidophilus*, *L. plantarum*, *L. paracasei*, *L. delbrueckii* subsp. *Bulgaricus*, *Bifidobacterium,* prebiotic fiber (fermented milk with prebiotic fiber)	125 g of fermented milk; 250 × 10^9^ CFU of probiotics; Once daily for 4 weeks	The frequency of complete bowel movements was increased.	[253]
PD patients	50–80 years	*n* = 50 (*n* = 25 received probiotics; *n* = 25 placebo)	*L. acidophilus*, *B. bifidum*, *L. reuteri*, *L. fermentum*	(2 × 10^9^ CFU for each strain) 8 × 10^9^ CFU per capsule; One capsule per day for 12 weeks	The expression of IL-1, IL-8, TNF-α, TGF-β, and PPAR-γ were improved. No change in inflammation and oxidative stress marker.	[254]

PD: Parkinson’s disease; *n*: number; CFU: colony-forming unit; GI-NMS: gastrointestinal-non-motor symptoms; PBMCs: peripheral blood mononuclear cells; hsCRP: high-sensitivity C-reactive protein; MDA: malondialdehyde; GSH: glutathione; MDS-UPDRS: Movement Disorders Society-Unified Parkinson’s Disease Rating Scale; TNF-α: tumor necrosis factor-alpha; IL-1: interleukin-1; IL-6: interleukin-6; IL-8: interleukin-8; IL-17A: interleukin-17A; TGF-β: transforming growth factor-beta; PPAR-γ: peroxisome proliferator-activated receptor-gamma; FOS: fructo-oligosaccharide. The studies proved that probiotics could be used to manage and treat several neuropsychiatric disorders and GI disorders [246]. Supplementing probiotics or fermented foods rich in probiotics could improve mental health and cognitive function and prevent respiratory infections in humans [255,256,257]. It is known that TLRs signaling is crucial for PD (refer to Section 6.1), and any modulations in its function using probiotics can regulate PD. In the significant TLR signaling, the TLR1, TLR2, and TLR 6 were modulated by the probiotics such as *L. rhamnosus* (JB-1), *L. casei Shirota*, and *L. reuteri* [121,243,247,258].

## Data Availability

The data presented in this study are available within the article.
